# Mapping Indigenous land management for threatened species conservation: An Australian case-study

**DOI:** 10.1371/journal.pone.0173876

**Published:** 2017-03-14

**Authors:** Anna R. Renwick, Catherine J. Robinson, Stephen T. Garnett, Ian Leiper, Hugh P. Possingham, Josie Carwardine

**Affiliations:** 1 ARC Centre of Excellence for Environmental Decisions, the NERP Environmental Decisions Hub, Centre for Biodiversity & Conservation Science, University of Queensland, Brisbane, Queensland, Australia; 2 CSIRO Ecosystem Sciences, Ecoscience Precinct, Brisbane, Queensland, Australia; 3 Northern Institute, Charles Darwin University, Darwin, Northern Territory, Australia; 4 Research Institute for the Environment and Livelihoods, Charles Darwin University, Darwin, Northern Territory, Australia; University of Porto, PORTUGAL

## Abstract

Much biodiversity lives on lands to which Indigenous people retain strong legal and management rights. However this is rarely quantified. Here we provide the first quantitative overview of the importance of Indigenous land for a critical and vulnerable part of biodiversity, threatened species, using the continent of Australia as a case study. We find that three quarters of Australia’s 272 terrestrial or freshwater vertebrate species listed as threatened under national legislation have projected ranges that overlap Indigenous lands. On average this overlap represents 45% of the range of each threatened species while Indigenous land is 52% of the country. Hotspots where multiple threatened species ranges overlap occur predominantly in coastal Northern Australia. Our analysis quantifies the vast potential of Indigenous land in Australia for contributing to national level conservation goals, and identifies the main land management arrangements available to Indigenous people which may enable them to deliver those goals should they choose to do so.

## Introduction

The past century has seen a significant rise in the recognition of Indigenous rights to land, leading to formal changes in the way land is governed and managed [1,2]. Much of the land that is owned and managed by Indigenous people is in places with high species richness and ecological intactness compared with more developed, modified and heavily populated areas [3,4]. Further, it is acknowledged that the biodiversity that occurs on Indigenous lands is highly dependent on Indigenous peoples’ knowledge, practices and cultural connections to their traditional estates [5,6]. Despite a history of intermittent conflict with the form of conservation imposed by settler societies [7,8], Indigenous communities frequently manage their land in ways that are not only consistent with biodiversity conservation but often have the explicit purpose of retaining it [9,10]. This provides benefits to broader society, including the protection of native flora and fauna, carbon sequestration and intact waterways [11], and these benefits are linked to human wellbeing [12].

The biodiversity values delivered by Indigenous lands are extremely important, particularly given current unprecedented global declines in species populations and ecosystem integrity [13]. The IUCN now classifies more than 20,000 plants and 57,000 animals as ‘threatened’ worldwide [14], with habitat loss, invasive species, overexploitation, pollution and nutrient loading and climate change identified as the main culprits [15]. The Convention on Biological Diversity (CBD) outlines a set of goals, including a target to prevent extinctions of threatened species and improve and sustain their conservation status (Aichi Target 12) [16]. Signatory nations translate these targets to national level strategies. In Australia, these include a national reserve system strategy (including Indigenous Protected Areas) that systematically conserves representative samples of biodiversity [17] and a national threatened species strategy aimed at halting species declines [18].

The implementation of Australia’s national level strategies requires conservation that transcends land tenure boundaries [19] and can be operationalized under a range of governance and implementation models, including the establishment of new protected areas and supporting community-based conservation on Indigenous land [20,21]. Initiatives that recognise and remunerate maintenance of the ecological values and services delivered from land managed by Indigenous people can serve both to protect nature and enhance the livelihoods of Indigenous people, many of whom are among the most disadvantaged in the world [8]. These initiatives include the establishment of Indigenous Protected Areas [22], Indigenous Community Conservation Areas (http://www.iccaconsortium.org/), and REDD+ and other Payments for Ecosystem Service schemes [23,24], as well as development projects which aim to integrate conservation and development for Indigenous peoples.

Given this context, it is perhaps unsurprising that Indigenous land management is a small but growing sector in many parts of the world [25]. In Australia, for example, there are now over 700 Indigenous land management rangers employed across the continent whose jobs and activities are predominantly financed by the Australian Government with an investment of approximately AU$85m per annum [26].The establishment of schemes to formalise Indigenous land management and the delivery of benefits to broader society have typically occurred based on local capacity, values and funding availability. As yet there is a lack of quantitative spatial understanding of how local-scale Indigenous Land and Sea Management (ILSM) efforts can link to national level strategic planning goals. A more strategic approach could ensure that the benefits of such schemes are maximised, for both Indigenous people and broader society, and that resources are allocated effectively, efficiently and equitably [27,28]. Information on the overlap between areas where and how Indigenous peoples can practice land management and the biodiversity that wider society values is a critical first step in identifying viable options for partnerships that maximise these outcomes.

In this paper we investigate the potential for threatened species management on Indigenous land, using the vast country of Australia as a case study. We use the distributions of threatened native vertebrate species to estimate the relative importance of Indigenous land in Australia for managing threatened species. We analyse overlap in modelled ranges for 272 terrestrial and freshwater wildlife species (includes subspecies) protected under national legislation (for all species and separate taxonomic groups) with the 52% of the Australian land mass to which an ongoing connection by Indigenous people is currently recognised. We investigate bioregional hotspots on Indigenous lands where the ranges of multiple threatened species ranges overlap. We then identify potential areas and pathways for Indigenous people to contribute to national level biodiversity priorities on their land should they choose to do so.

## Methods

We created a map of Indigenous land across Australia using data from official agencies responsible for the registration of various Indigenous land tenures. These include National Native Title Tribunal, Australian Government Department of Environment and Energy, Indigenous Land Corporation, National Land and Water Resources Audit, and Geoscience Australia. The map represents legally recognised tenure with the type of rights and opportunities conveyed varying with the form of tenure. We categorized the different Indigenous land management pathways according to the rights Indigenous people have over land management under Australian law into those areas where Indigenous people have freehold land tenure and exclusive native title rights (113.59 million hectares Mha), Indigenous people have established co-management partnerships in protected areas (27.36 Mha), and tenure where Indigenous people have negotiated to take part in conservation management as part of a land use agreement (259.75 Mha) (Table 1).

**Table 1:** Indigenous tenure layers categorised into the types of land management rights available.

Indigenous land management category	Indigenous tenure layers	Source	Area and % of all Indigenous land
Indigenous people have freehold land tenure and exclusive native title rights	Indigenous Land Tenure 1994 (freehold, leasehold or reserve 100km2)	Australian Land Tenure 1993 –Version 3 (2004), National Mapping Division, Geosciences Australia.	113.59 Mha (28.34%)
	Indigenous Land Cooperation land	PSMA Australian National Land Tenure Classification Version 1.2 (2008), PSMA Australia Limited.	
	Aboriginal freehold land at Olkola and Kalpower, Queensland	Digital Cadastre DataBase (DCDB), Department of Natural Resources and Mines, Queensland Government [accessed 12 December 2015 from Queensland Spatial Catalogue]	
Indigenous people have established co-management partnerships in protected areas	Indigenous Protected Areas Declared 2015 (Declared)	Environmental Resources Information Network, Department of the Prime Minister and Cabinet and Department of the Environment, Commonwealth of Australia 2015	27.36 Mha (6.83%)
	Collaborative Australian Protected Areas Database 2014 (Aboriginal areas and Joint National Parks)		
	Indigenous Land Tenure 1993 (freehold-national Parks)	Australian Land Tenure 1993 –Version 3 (2004), National Mapping Division, Geosciences Australia.	
Indigenous people have negotiated to take part in conservation management as part of a land use agreement	Indigenous Land Use Agreements (Registered)	National Native Title Tribunal, Commonwealth of Austral	259.75 Mha (64.81%)
	Register of Native Title Claims (Native Title exists)		

We utilized distribution data of Australian vertebrate fauna (mammals, birds, reptiles, frogs and fish) listed as threatened under the Environmental Protection and Biodiversity Conservation Act 1999 (EPBC Act) from the Species of National Environmental Significance Database. These maps are predictive distributions of individual species based on range and suitable habitat. The precision of the data is divided into three classes: 1) Known to occur–areas of preferred habitat near known locations, 2) Likely to occur–areas of preferred habitat within the range of the species, and 3) May occur–areas within a broad environmental envelope or geographic region that encompasses the probable range of the species. In our analyses we only included data from the first two categories. These were amalgamated into a single class.

Species distributions were generalised to a 10km^2^ grid resolution (0.1 degrees). Migratory and marine species were removed as the distributions available may not reflect the total distribution of these species, leaving a total of 272 species for which we have distribution data (80 bird species, 40 fish, 28 frogs, 79 mammals and 45 reptiles). Species were grouped into their scheduled threat status within the EPBC Act List of Threatened Species (Critically Endangered, Endangered, and Vulnerable).

### Analysis

We created a map of area covering the combined modelled ranges of the 272 threatened species across Australia and overlaid this with the map of Indigenous land. From this we determined the number of species with ranges that overlap with Indigenous lands (and within each of the Indigenous land management categories) as well as the proportion of each species range that overlaps for all threatened species, species in each taxonomic group and the 40 priority species from the National Threatened Species Strategy). We then determined the number of species (overall and in each taxonomic group) occupying each 10km^2^ across Indigenous lands using the ‘Count Overlap Generic tool’ in ArcMap. We created a bioregion ‘hotspot’ map, using the Interim Biogeographic Regionalisation for Australia data [29], of the total number of overlapping threatened species in each 10km^2^ across the Indigenous land summed within each bioregion. We classified ‘hotspots’ as the bioregions where the greatest number of threatened species have overlapping ranges. The number of species ranges within each 10km2 grid cell in each bioregion are summed. Species ranges that cover more than 10km^2^ are counted in each 10km^2^ grid cell they occupy. All analysis was carried out in ArcMap 10.3.1.

## Results

**Fig 1:**
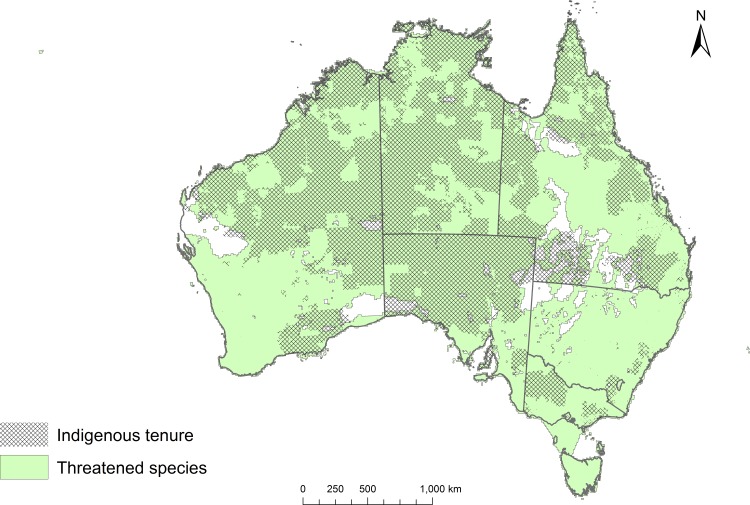
Combined extent of threatened vertebrate species habitat and the overlap with Indigenous land across Australia.

Australia’s terrestrial land area covers approximately 769 Mha, of which 401 Mha (or 52%) is covered by some form of Indigenous tenure (Fig 1). The total area of combined habitat ranges occupied by all 272 threatened species is 735Mha. Of this, 51.3% (376.9Mha) is on Indigenous land, ranging from 57.4% for mammals to 18.1% for fish (Table 1). Almost three quarters, 74.3% (202), of Australian threatened species have at least part of their modelled range on Indigenous land. The percentage of species ranges on Indigenous land for frogs is 85.7%, for mammals 82.3%, and is lowest for fish (57.5%) and reptiles (68.9%) (Table 1). Twenty-two species have more than 75% of their range on Indigenous land (Table 2) including five species with more than 99% of their range on Indigenous land (*Amytornis merrotsyi pedleri* Gawler Rangers Short-tailed Grasswren, *Lasiorhinus krefftii* Northern Hairy-nosed Wombat,*Mogurnda clivicola* Flinders Ranges Mogurnda, *Zyzomys maini* Arnhem Land Rock Rat and *Zyzomys palatalis* Carpentarian Rock Rat). At least 31of the 20 birds and 20 mammals listed for special attention under the National Threatened Species Strategy occur on Indigenous lands. Over 70% of the projected range of the *Lagorchestes hirsutus* Mala, *Zyzomys pedunculatus* Central Rock Rat, *Notomys aquilo* Northern Hopping Mouse, *Pezoporus occidentalis* Night Parrot and *Amytornis woodwardi* White-throated Grasswren is encompassed within Indigenous lands.

**Table 2:** Proportion and extent of the overlap in the habitat ranges of threatened vertebrate species taxonomic groups with Indigenous land in Australia.

Taxonomic Group	Percentage (and area) of range on Indigenous land	Percentage (and #) of species with at least some range on Indigenous land
All	51.3 (376.9Mha)	74.3 (202)
Birds	42.9 (215.0Mha)	73.8 (59)
Fishes	18.1 (6.9Mha)	57.5 (23)
Frogs	23.1 (12.8Mha)	85.7 (24)
Mammals	57.4 (301.9Mha)	82.3 (65)
Reptiles	43.3 (51.6Mha)	68.9 (31)

**Fig 2:**
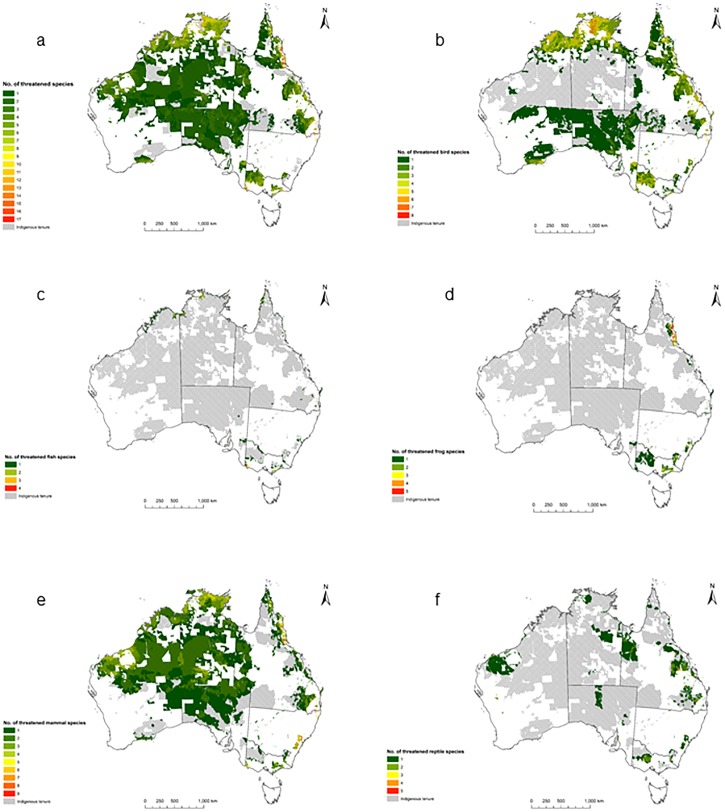
Number of threatened species within each 10km^2^ of Indigenous land across Australia: a) all species, b) birds, c) fish, d) frogs, e) mammals, and f) reptiles.

The number of species in any 10km^2^ cell ranged from one to 17. The range for each of the individual species groups was from one to nine for mammals, one to eight for birds, one to five for frogs and reptiles, and one to four for fishes. For all species, apart from reptiles, the coastal areas of Indigenous land contained the greatest number of species (Fig 2). By far the greatest number of overlapping species ranges on the Indigenous land within the 89 bioregions were in South East Queensland (1486 10km^2^ grid cells containing overlapping species ranges, each with a minimum of three and maximum of 17 overlapping species ranges totalling 16534 overlapping species ranges across the bioregion), Southern Coastal Plains (1866 10km^2^ grid cells containing overlapping species ranges, each with a minimum of two and maximum of 14 overlapping species ranges totalling 15691 overlapping species ranges across the bioregion) and Northern Kimberley (2261 10km^2^ grid cells containing overlapping species ranges, each with a minimum of one and maximum of nine overlapping species ranges totalling 12911 overlapping species ranges across the bioregion) (S1 Table).

**Fig 3:**
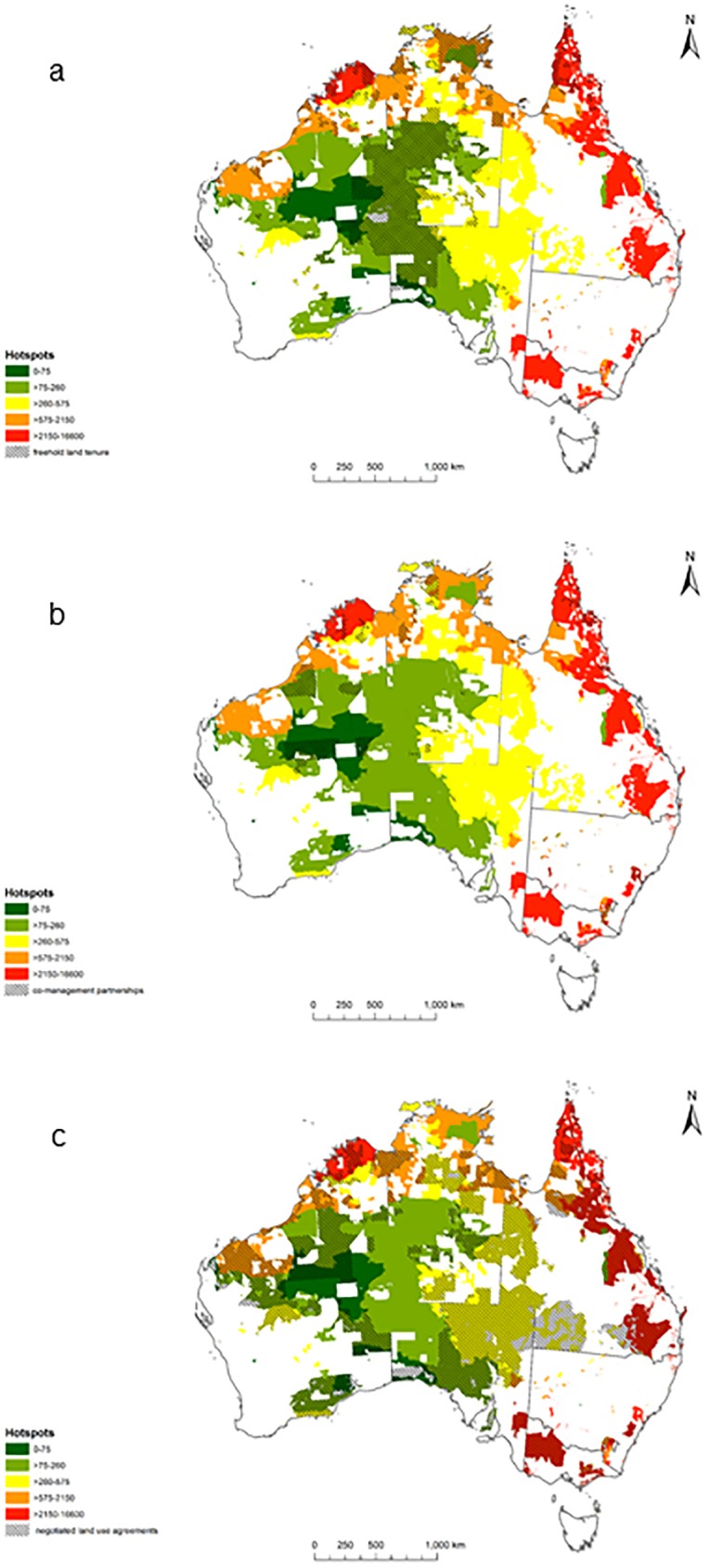
Overlap of hotspots with indigenous land tenure categories: a) freehold land tenure b) co-management partnerships c) negotiated land use agreement.

Areas of Indigenous land where Indigenous people have freehold land tenure and exclusive native title rights contain at least part of the range of 51% of threatened species. Areas where Indigenous people have established conservation co-management partnerships only contain ranges of 4% of threatened species. Areas where Indigenous people have negotiated to take part in conservation management contain ranges of 32% of threatened species (Fig 3).

## Discussion

Indigenous lands support a high proportion of Australia’s threatened species with some hotspots, particularly in coastal areas and in northern Australia, supporting up to 17 threatened vertebrates. For many threatened vertebrate species Indigenous land is essential for their persistence. This is even more notable when including not only those that occur predominantly on Indigenous lands but also those for with only a small part of their range on Indigenous land but for which conservation opportunities in other parts of their range are hindered by a multitude of threats and activities. The overlap is particularly high for mammals and birds listed in the National Threatened Species Strategy, which emphasises the importance of the potential role of Indigenous people in threatened species conservation. Furthermore, these estimates are a minimum and Indigenous people also assert ongoing connection to many areas for which claims are pending or not yet lodged.

Australia has been identified as having one of the highest levels of correlation between cultural and biological diversity of any country in the world [30]. Indigenous land in Australia harbours almost an equal proportion, on a per hectare basis, of the ranges of threatened vertebrate species as non-Indigenous land, i.e. 52% of land is Indigenous, and 52% of threatened vertebrate species habitat ranges occur on Indigenous lands. Indigenous lands have not been subject to intensive development, but a combination of natural rarity and specialisation in species, the spread of invasive species and changes in fire management have nevertheless rendered many of the species that live there threatened. Importantly, not one of the species considered is threatened by current or traditional Indigenous land management practices.

As a nation, Australia is globally remarkable for its high levels of recent extinctions and declines in native species [31], but also for its capacity to address these declines [32]. Australia’s national strategies are testament to efforts to protect land and manage habitat to avoid species extinctions. However, many threatened species are not well-represented in Australia’s current protected area network [33], and much available funding for threatened species conservation is yet to be translated to on-ground actions. We show that Indigenous land within the bioregions around the coastal areas of northern, eastern and southern Australia are of high importance for threatened species.

Our analysis provides a quantitative overview of where Indigenous peoples’ work on threatened vertebrates may contribute to achieving national strategies. Our results show that over 50% of threatened vertebrate species in Australia occur in areas where Indigenous people have exclusive land rights. Hotspots identified include South Eastern Queensland; South East Coastal Plains; and Northern Kimberley bioregions. This analysis also shows that 32% of threatened vertebrate species are being managed on Indigenous lands under conservation co-management agreements, suggesting that this pathway remains an attractive option for Indigenous people who wish to engage in conservation agreements. Importantly these agreements would need to align with Indigenous and threatened species conservation goals and ensure Indigenous benefits are appropriately considered in payment for environmental service or collaborative management agreements. In some cases successful agreements may not be just in threatened species hotspots but where multiple conservation and Indigenous community benefits can be negotiated and delivered [34].

Importantly this analysis highlights the need for an intercultural approach to threatened species conservation in countries such as Australia. Based on rights, Indigenous people need to help guide appropriate goals and strategies to develop a shared understanding of what threatened species conservation entails, how it can be negotiated, delivered and evaluated, and how different interests in the benefits of threatened species conservation can be reconciled. The information provided by this research can be part of the evidence base to assist Indigenous people in gaining support for threatened species conservation that can align with their own local priorities, including opportunities to expand their portfolio of income sources, should they wish to do so. This information can also assist policy makers to engage strategically with the Indigenous peoples whose land supports large numbers of threatened species or species considered of particularly high conservation value, and to guide funding programs focused on supporting Indigenous land management activities (e.g. Robinson et al., 2016).

This analysis shows which areas capture some valued features of a landscape i.e. threatened species. This is only the first stage of a strategic assessment of opportunities for Indigenous engagement with threatened species conservation. We acknowledge that areas and opportunities for Australia’s ecological values extend further than threatened species, and do not claim to have captured all potential opportunities. Further efforts are also required to understand how locally-held conservation values of Indigenous people align with national-level goals. It is likely that some trade-offs as well as synergies exist, with some species and ecological processes more important to local Indigenous people, and some prioritised higher at the national level. In addition to this, there are likely to be both trade-offs and opportunities among the goals of biodiversity conservation, livelihoods and the priorities of local communities who own the lands where threatened species exist.

Conservation standards and guidelines are now emerging which take the social, economic and cultural impacts of conservation and Indigenous rights into account [35,36]. The principle of common but differentiated responsibility describes that contributions to high level conservation goals should be fair and equitable [27,28]. We need to consider the extent to which Indigenous people are expected to sustain our planet’s natural systems, the consequences of this potentially inequitable expectation of responsibility, and whether they are sufficiently empowered and resourced to decide how they choose to respond to wider expectations.

## Supporting information

S1 Table Total number of overlapping threatened vertebrate species habitat ranges in each 10km^2^ across Indigenous lands summed within each of the 89 bioregions (species ranges that cover more than 10km^2^ are counted in each 10km^2^ grid cell they occupy). (DOCX)
