# Supplementary material for: Mapping Indigenous land management for threatened species conservation: An Australian case-study
Source: PLoS One. 2017 Mar 14;12(3):e0173876. doi: 10.1371/journal.pone.0173876 (PMC5349676; doi:10.1371/journal.pone.0173876)
Supplement: S1 Table — (DOCX) [file pone.0173876.s001.docx]

Supporting Information

S1 Table. Total number of overlapping threatened vertebrate species habitat ranges in each 10km^2^ across Indigenous lands summed within each of the 89 bioregions (species ranges that cover more than 10km^2^ are counted in each 10km^2^ grid cell they occupy).

| **IBRA** | **IBRA Full name** | | **Total number of overlapping threatened vertebrate species habitat ranges in each 10km^2^ of Indigenous tenure** |
| --- | --- | --- | --- |
| SEQ | South Eastern Queensland | 16534 | |
| SCP | South East Coastal Plain | 15691 | |
| NOK | Northern Kimberley | 12911 | |
| WET | Wet Tropics | 9198 | |
| SEH | South Eastern Highlands | 6710 | |
| SVP | Southern Volcanic Plain | 6535 | |
| SYB | Sydney Basin | 5694 | |
| SEC | South East Corner | 5596 | |
| BBN | Brigalow Bell North | 3474 | |
| RIV | Riverina | 3038 | |
| BBS | Brigalow Belt South | 2899 | |
| VIM | Victoria Minlands | 2821 | |
| MDD | Murray Darling Ranges | 2794 | |
| CYP | Cape York Peninsula | 2783 | |
| NCP | Naracoorte Coastal Plain | 2723 | |
| EIU | Einasleigh Uplands | 2156 | |
| VIB | Victoria Bonaparte | 1915 | |
| ARC | Arnhem Coast | 1630 | |
| DAC | Darwin Coastal | 1579 | |
| PIL | Pilbara | 1515 | |
| NNC | NSW North Coast | 1381 | |
| BHC | Broken Hill Complex | 1271 | |
| GUC | Gulf Coastal | 1178 | |
| DAL | Dampierland | 1145 | |
| AUA | Australian Alps | 1096 | |
| GFU | Gulf Fall and Uplands | 1001 | |
| GUP | Gulf Plains | 776 | |
| DRP | Darling Riverine Plains | 773 | |
| NSS | NSW South Western Slopes | 706 | |
| OVP | Old Victoria Plain | 630 | |
| ARP | Arnhem Plateau | 588 | |
| TIW | Tiwi Cobourg | 575 | |
| CEK | Central Kimberley | 559 | |
| MAC | MacDonnell Ranges | 512 | |
| PCK | Pine Creek | 482 | |
| CMC | Central Mackay Coast | 479 | |
| FLB | Flinders Lofty Block | 465 | |
| CHC | Channel Country | 403 | |
| NET | New England Tablelands | 362 | |
| ESP | Esperance Plains | 350 | |
| MGD | Mitchell Grass Downs | 343 | |
| FIN | Finke | 335 | |
| MUL | Mulga Lands | 317 | |
| MII | Mount Isa Inlier | 308 | |
| STU | Sturt Plateau | 299 | |
| STP | Stony Plains | 292 | |
| MUR | Murchison | 278 | |
| SSD | Simpson Strzelecki Dunefields | 262 | |
| GSD | Great Sandy Desert | 214 | |
| CEA | Central Arnhem | 204 | |
| DAB | Daly Basin | 189 | |
| MAL | Mallee | 179 | |
| DEU | Desert Uplands | 175 | |
| GAS | Gascoyne | 168 | |
| GVD | Great Victoria Desert | 162 | |
| GAW | Gawler | 155 | |
| CER | Central Ranges | 153 | |
| EYB | Eyre Yorke Block | 134 | |
| COO | Coolgardie | 126 | |
| DMR | Davenport Murchison Ranges | 113 | |
| TAN | Tanami | 111 | |
| BRT | Burt Plain | 102 | |
| NAN | Nandewar | 93 | |
| FUR | Furneaux | 76 | |
| TSE | Tasmanian South East | 60 | |
| SWA | Swan Coastal Plain | 56 | |
| NUL | Nullarbor | 50 | |
| LSD | Little Sandy Desert | 49 | |
| KIN | King | 45 | |
| AVW | Avon Wheatbelt | 40 | |
| CAR | Carnarvon | 35 | |
| JAF | Jarrah Forest | 34 | |
| GID | Gibson Desert | 30 | |
| COP | Cobar Peneplain | 27 | |
| HAM | Hampton | 20 | |
| GES | Geraldton Sandplains | 4 | |
| KAN | Kanmantoo | 4 | |
| WAR | Warren | 4 | |
| YAL | Yalgoo | 3 | |
| COS | Coral Sea | 0 | |
| PSI | Pacific Subtropical Islands | 0 | |
| SAI | Subantarctic Islands | 0 | |
| TWE | Tasmanian West | 0 | |
| BEL | Ben Lomond | 0 | |
| ITI | Indian Tropical Islands | 0 | |
| TCH | Tasmanian Central Highlands | 0 | |
| TNM | Tasmanian Northern Midlands | 0 | |
| TNS | Tasmanian Northern Slopes | 0 | |
| TSR | Tasmanian Southern Ranges | 0 | |
